# The need to adopt the z-score in the interpretation of spirometry in Brazil

**DOI:** 10.36416/1806-3756/e20260011

**Published:** 2026-05-22

**Authors:** Maurício Domingues Ferreira

**Affiliations:** 1. Laboratório de Investigação Médica em Imunologia - LIM-56 - Instituto de Medicina Tropical Medicine, Faculdade de Medicina, Universidade de São Paulo - FMUSP - São Paulo (SP) Brasil.

## DEAR EDITOR:

The interpretation of pulmonary function tests is central to the diagnosis and prognostic stratification of respiratory diseases. In Brazil, however, the use of percent predicted values and fixed cut-off points, such as an FEV_1_/FVC ratio < 0.70, still predominates, despite well-recognized statistical limitations. These criteria disregard the physiological variability of lung function across the lifespan and result in systematic misclassification. In this context, the adoption of the z-score and statistically defined limits of normality, as proposed by the Global Lung Function Initiative (GLI) equations^1^ and the 2024 Brazilian spirometry recommendations,^2^ represents an essential methodological advance. The present letter critically discusses the persistent limitations of traditional interpretative methods and emphasizes the need for the systematic incorporation of the z-score into national clinical practice.

The use of percent predicted values derives from their apparent simplicity but overlooks a fundamental aspect: these values do not follow a normal distribution. The same “80% of predicted” may correspond to markedly different degrees of functional impairment in individuals of different ages, heights, or ethnic backgrounds. In practice, this leads to a dual pattern of error: the overdiagnosis of airflow obstruction in older adults due to the physiological age-related decline in the FEV_1_/FVC ratio, and its underdiagnosis in younger adults, whose greater functional reserve may mask early disease. This phenomenon was clearly demonstrated by Miller et al.,^3^ who identified misclassification in more than 20% of patients when percent predicted values and fixed thresholds were used as interpretative criteria.

The GLI 2012 equations^1^ introduced a decisive methodological refinement by incorporating the LMS (lambda-mu-sigma) model, which estimates the mean, variability, and skewness of lung function in large healthy populations. This approach enabled the generation of continuous reference values from 3 to 95 years of age, simultaneously adjusted for age, sex, height, and ethnicity, consolidating the z-score as the most statistically coherent metric for defining normality. The 2024 Brazilian spirometry recommendations,^2^ aligned with those by ATS/ERS^4^ and the most recent international standards, establish that the lower limit of normal (LLN) should be defined by the 5th percentile (z < −1.64), explicitly recommending the abandonment of fixed cut-offs and percent predicted values as primary interpretative criteria.

The z-score also modernizes the assessment of lung volumes and diffusing capacity. GLI reference equations for lung volumes and transfer lung capacity for carbon monoxide (TL_CO_)^5^ have improved internal consistency across parameters and enabled more accurate detection of functional impairment in patient groups that were previously underrecognized. Small reductions in TL_CO_ z-scores, for example, may reflect clinically meaningful declines that would be overlooked if only percent predicted values were considered, particularly in older individuals or in those at the extremes of height. This methodological difference has direct implications in clinical settings such as interstitial lung diseases, pulmonary hypertension, and combined respiratory disorders.

Neder et al. further emphasize that appropriate spirometric interpretation should be based on z-scores, as this metric reflects the deviation of measured values relative to the true variability of the reference population, adjusted for age, sex, and height. They highlight that percent predicted values may lead to erroneous interpretations, especially at the extremes of age, and stress that statistical limits of normality do not represent rigid boundaries between health and disease, but rather should be interpreted in light of the clinical context and pre-test probability. These findings consolidate the z-score as the most appropriate metric for a statistically sound and clinically informed interpretation of pulmonary function tests.^6^

The ATS/ERS interpretative update^4^ reinforced this paradigm by establishing that spirometric abnormalities should be defined exclusively using z-scores. In addition, Almeshari et al.^7^ demonstrated that interpreting mid-expiratory flow (FEF_25-75%_) using z-scores significantly improves the detection of early small airways dysfunction, further underscoring the direct clinical impact of adopting this metric for the early identification of bronchiolar abnormalities.

Diagnostic accuracy is also intrinsically linked to equity. Moffett et al.^8^ showed that race-neutral reference equations, when interpreted using z-scores, reduce biases associated with race-specific equations and improve comparability across patients. In line with these findings, Brems et al.,^9^ analyzing more than 13,000 patients with COPD, demonstrated that the use of percent predicted values resulted in unequal misclassification between Black and White individuals (20.2% vs. 6.1%), whereas interpretation based on z-scores eliminated this disparity (12.6% vs. 12.3%). Importantly, only z-score-based classifications were significantly associated with clinically meaningful risk, showing a robust association between increasing severity and exacerbation risk (OR = 2.34; 95% CI, 1.51-3.63). Percent predicted-based classifications, in contrast, were not significantly associated with clinical outcomes.

In interstitial lung diseases, contemporary evidence indicates that the adoption of z-scores is even more critical. The largest study conducted to date in this field, involving 6,808 patients, by Boros et al.^10^ demonstrated that z-score-based criteria led to the reclassification of approximately one-quarter of patients, with direct implications for therapeutic decision-making. Each one-unit reduction in FVC z-score was associated with a 10.3% increase in mortality risk, whereas equivalent reductions in TL_CO_ z-score increased this risk by more than 30%. These findings indicate that interpretation based exclusively on percent predicted values tends to underestimate functional severity and may delay essential clinical interventions, such as early initiation of antifibrotic therapy, intensified monitoring, and timely referral for lung transplantation evaluation.

Similar results have been observed in large population-based cohorts. Cestelli et al.^11^ showed that modest reductions in FEV_1_, FVC, and TL_CO_ z-scores are consistently associated with all-cause and respiratory mortality, indicating that z-scores capture true clinical risk, whereas percent predicted values often mask functionally relevant declines. Together, these data reinforce that traditional interpretative methods may distort diagnoses and compromise clinical decision-making. The 2024 Brazilian spirometry recommendations^2^ explicitly acknowledge these limitations and advise to modernize functional interpretation, positioning the adoption of z-scores as a methodological advance aligned with international best practices, with direct implications for diagnostic accuracy, equity, and patient safety.
